# Alleles of the homologous recombination gene, *RAD59,* identify multiple responses to disrupted DNA replication in *Saccharomyces cerevisiae*

**DOI:** 10.1186/1471-2180-13-229

**Published:** 2013-10-14

**Authors:** Lauren C Liddell, Glenn M Manthey, Shannon N Owens, Becky XH Fu, Adam M Bailis

**Affiliations:** 1 Department of Molecular and Cellular Biology, Beckman Research Institute of the City of Hope, 91010 Duarte, CA, USA; 2 Irell & Manella Graduate School of Biological Sciences, Beckman Research Institute of the City of Hope, 91010 Duarte, CA, USA; 3 Occidental College, 90041 Los Angeles, CA, USA; 4 Department of Genetics, Stanford University School of Medicine, 94305 Stanford, CA, USA

**Keywords:** Homologous recombination, *Saccharomyces cerevisiae*, DNA replication, Genome stability, Loss of heterozygosity

## Abstract

**Background:**

In *Saccharomyces cerevisiae*, Rad59 is required for multiple homologous recombination mechanisms and viability in DNA replication-defective *rad27* mutant cells. Recently, four *rad59* missense alleles were found to have distinct effects on homologous recombination that are consistent with separation-of-function mutations. The *rad59-K166A* allele alters an amino acid in a conserved α-helical domain, and, like the *rad59* null allele diminishes association of Rad52 with double-strand breaks. The *rad59-K174A* and *rad59-F180A* alleles alter amino acids in the same domain and have genetically similar effects on homologous recombination. The *rad59-Y92A* allele alters a conserved amino acid in a separate domain, has genetically distinct effects on homologous recombination, and does not diminish association of Rad52 with double-strand breaks.

**Results:**

In this study, *rad59* mutant strains were crossed with a *rad27* null mutant to examine the effects of the *rad59* alleles on the link between viability, growth and the stimulation of homologous recombination in replication-defective cells. Like the *rad59* null allele, *rad59-K166A* was synthetically lethal in combination with *rad27*. The *rad59-K174A* and *rad59-F180A* alleles were not synthetically lethal in combination with *rad27,* had effects on growth that coincided with decreased ectopic gene conversion, but did not affect mutation, unequal sister-chromatid recombination, or loss of heterozygosity. The *rad59-Y92A* allele was not synthetically lethal when combined with *rad27*, stimulated ectopic gene conversion and heteroallelic recombination independently from *rad27*, and was mutually epistatic with *srs2*. Unlike *rad27*, the stimulatory effect of *rad59-Y92A* on homologous recombination was not accompanied by effects on growth rate, cell cycle distribution, mutation, unequal sister-chromatid recombination, or loss of heterozygosity.

**Conclusions:**

The synthetic lethality conferred by *rad59* null and *rad59-K166A* alleles correlates with their inhibitory effect on association of Rad52 with double-strand breaks, suggesting that this may be essential for rescuing replication lesions in *rad27* mutant cells. The *rad59-K174A* and *rad59-F180A* alleles may fractionally reduce this same function, which proportionally reduced repair of replication lesions by homologous recombination and growth rate. In contrast, *rad59-Y92A* stimulates homologous recombination, perhaps by affecting association of replication lesions with the Rad51 recombinase. This suggests that Rad59 influences the rescue of replication lesions by multiple recombination factors.

## Background

In *Saccharomyces cerevisiae*, defective DNA replication stimulates homologous recombination (HR), suggesting that the lesions that accumulate following replication failure are substrates for HR [1-11]. Rad27 is a structure-specific endonuclease [12] required for completion of lagging strand synthesis [13], and has also been implicated in base excision repair [14], and double-strand break repair by non-homologous end joining [15]. Loss of Rad27 leads to accumulation of single-stranded gaps or nicks on daughter DNA strands [2,16]. Collision of replication forks with these lesions results in fork collapse and generation of double-strand breaks (DSB) [8,17] that can stimulate HR. Importantly, concomitant loss of Rad27 and components of the HR apparatus leads to synthetic lethality [18-20]. These observations implicate HR in repair of DSBs that accumulate in the absence of Rad27. Failure to repair DSBs leads to chromosome loss [21] that is greatly stimulated in *rad27* null mutant cells [8], suggesting that the essential role for the HR apparatus in *rad27* mutants may be prevention of lethal levels of chromosome loss.

*RAD59* encodes a protein that augments the ability of Rad52, the central HR protein in yeast [22,23], to anneal complementary DNA strands *in vitro*[24], and both are required for viability in *rad27* null mutant cells [19,20]. *RAD59* and *RAD52* are also required to repair DSBs by single-strand annealing (SSA) [21,25-28], and HR between inverted repeats by an annealing-dependent template switch at stalled replication forks [29-31]. Since *RAD59* exerts much of its effect on HR with *RAD52*[21,32,33], the function of *RAD59* required in the absence of *RAD27* may be in collaboration with *RAD52*.

The purpose of the current study was to explore the function of *RAD59* required for the viability of *rad27* null mutant cells. We investigated how four *rad59* mutations previously characterized with respect to their effects on SSA [21,27], affected survivorship when combined with a *rad27* null mutation*.* We found that *rad59-K166A*, which alters an amino acid in a conserved, putative α-helical domain [27,34,35], was synthetically lethal in combination with *rad27*. Because *rad59-K166A* diminishes association of Rad52 with DSBs [21], this may be a function required for the viability of *rad27* null mutant cells. The *rad59-K174A* and *rad59-F180A* mutations, which alter amino acids in the same α-helical domain, and have genetically similar effects on SSA [21], were not synthetically lethal with *rad27*, but resulted in distinct effects on growth that correlated with their degree of inhibition of HR. This strongly implicates *RAD59-*dependent HR as a requirement for viability in *rad27* null mutant cells. The *rad59-Y92A* mutation, which alters an amino acid in a separate, conserved loop domain and confers genetically distinct effects on SSA [27,34] was not synthetically lethal with *rad27*, and had a stimulatory effect on HR. This effect was genetically equivalent to that of a null allele of *SRS2*, which encodes a helicase that disassembles Rad51-DNA filaments [36,37], suggesting that Rad59 may affect association of Rad51 with replication lesions. The distinct effects of the *rad59* alleles suggest that Rad59 possesses multiple, discrete roles in responding to the consequences of dysfunctional replication.

## Results

### The *rad59* mutant alleles display distinct effects on survival and growth in cells defective for lagging strand synthesis

**Figure 1:**
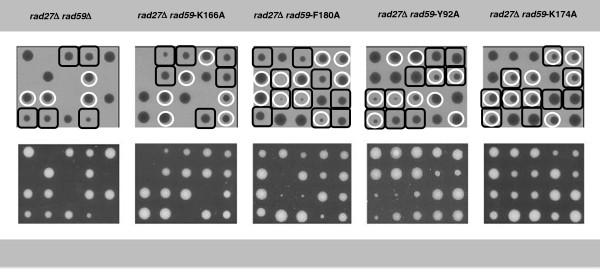
**The *rad59 *mutant alleles have distinct effects on survival in cells that are defective for lagging strand synthesis.** Diploid strains heterozygous at the *RAD27* (*rad27::LEU2*/*RAD27*) and *RAD59* (*rad59/RAD59*) loci were sporulated and tetrads dissected onto YPD medium. The resulting colonies were examined after 72 h of growth at 30°. Colonies from five representative tetrads from each strain are displayed. The genotype of each colony was determined by PCR as described in the Methods. In the inverted image, colonies possessing a *rad27::LEU2* allele are boxed in black, and those possessing a *rad59* allele are circled in white.

To further explore the function of *RAD59* required for viability in *rad27* null mutant cells, the effects of combining the *rad27::LEU2* allele with the various *rad59* alleles were determined by examining their ability to yield viable spores upon co-segregation in genetic crosses. The various *RAD27/rad27::LEU2 RAD59/rad59* double heterozygotes were sporulated and tetrads dissected onto rich medium (Figure 1). As observed previously, the *rad27::LEU2* and *rad59::LEU2* alleles did not appear together in any of the colonies arising from the spores, consistent with synthetic lethality [19,20]. The *rad59-K166A* allele, which alters a conserved lysine in the region of Rad59 that corresponds to the α-helical domain of the β − β − β − α motif of human Rad52 (Additional file 1: Figure S1) [27,34,35] displayed the same failure to appear with the *rad27::LEU2* allele, indicative of synthetic lethality.

**Figure 2:**
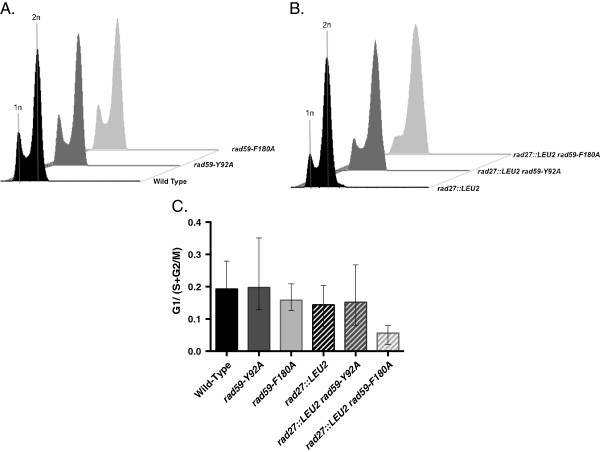
**The *rad59 *mutant alleles have distinct effects on cell cycle distribution in *rad27::LEU2 *mutant cells.** Wild-type, single and double mutant strains were grown to mid-log phase at 30°, fixed, stained with propidium iodide, and submitted to flow cytometric analysis as described in the Methods. **(A)** Cell cycle profiles for wild-type and *rad59* mutant strains. **(B)** Cell cycle profiles for *rad27* single and *rad27 rad59* double mutants. The distribution of cells with 1n and 2n DNA content in representative cultures of each strain are depicted. **(C)** Cell cycle distribution for wild-type and mutant strains. Median ratios of G1 to S + G2/M cells from a minimum of five independent cultures are indicated for each strain, and 95% confidence intervals are plotted.

The *rad59-K174A* and *rad59-F180A* alleles alter conserved amino acids in the same putative α-helical domain as *rad59-K166A* but were able to form viable spores upon segregation with *rad27::LEU2* (Figure 1). Doubling time of the *rad27::LEU2 rad59-F180A* double mutant was a statistically significant (p = 0.045) 24% longer than that observed for the *rad27* single mutant, which correlated with a ratio of G1 to S + G2/M cells that was a statistically significant (p = 0.0031) 2.6-fold lower (Figure 2; Additional file 1: Table S2). In contrast, doubling time of the *rad27::LEU2 rad59-K174A* double mutant was not significantly different from that of the *rad27::LEU2* single mutant (p = 0.71) (Table 1; Additional file 1: Table S2).

**Table 1:** Doubling times in wild-type and mutant haploid cells

**Genotype**	**Doubling time (min)**	**95% confidence interval**
Wild-type	111	99, 120
*rad59-Y92A*	119	97, 124
*rad59-K174A*	131	111, 147
*rad59-F180A*	112	99, 128
*rad27::LEU2*	164	137, 180
*rad27::LEU2 rad59-Y92A*	176	136, 195
*rad27::LEU2 rad59-K174A*	153	126, 177
*rad27::LEU2 rad59-F180A*	205	183, 230

Doubling times of freshly dissected segregants were determined as described in the Methods. Displayed for each genotype is the median doubling time and 95% confidence interval, determined from at least ten independent cultures.

The *rad59-Y92A* allele alters a conserved amino acid in another region of extensive conservation with Rad52 (Additional file 1: Figure S1) [27,34], and was observed to yield viable spores upon segregation with *rad27::LEU2* (Figure 1). While the colonies derived from the *rad27::LEU2 rad59-Y92A* double mutant spores sometimes appeared smaller than the *rad27::LEU2* single mutant colonies on dissection plates, neither the doubling times (p = 0.707) (Table 1; Additional file 1: Table S2), nor the ratios of G1 to S + G2/M cells (p = 0.60) (Figure 2, Additional file 1: Table S2) were significantly different for the *rad27::LEU2* single and *rad27::LEU2 rad59-Y92A* double mutant strains. This suggests that germination of *rad27::LEU2 rad59-Y92A* double mutant spores may sometimes take longer than *rad27::LEU2* single mutant spores. We did not observe significant effects of the tested *rad59* missense alleles on doubling time (p > 0.15) (Table 1; Additional file 1: Table S2), or cell cycle distribution (p > 0.50) (Figure 2; Additional file 1: Table S2) in cells that possessed a wild-type *RAD27* gene. Since all four *rad59* missense mutations support steady-state levels of Rad59 that are comparable to wild-type [27], their effects on viability and growth when combined with *rad27::LEU2* cannot be attributed to changes in the level of Rad59 in the cell. Altogether, these observations suggest that *RAD59* plays a critical role in determining the growth characteristics of cells defective for lagging strand synthesis.

### The *rad59* alleles affect a *RAD51*-dependent mechanism for repairing replication lesions

**Figure 3:**
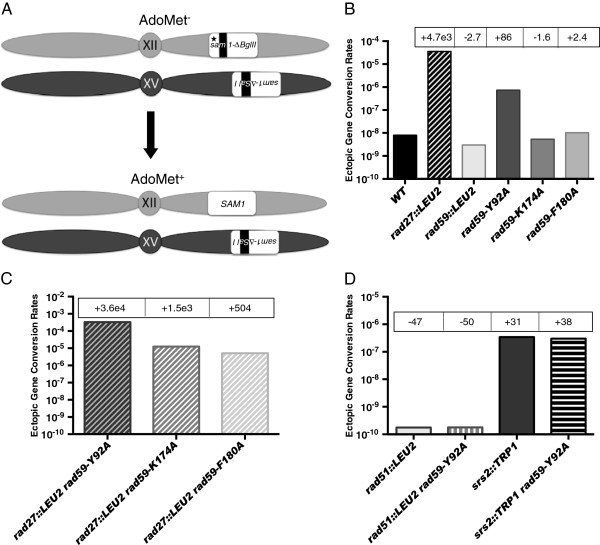
**The *rad59 *mutant alleles have distinct effects on gene conversion between un-linked repetitive elements in haploid strains. (A)** The spontaneous ectopic gene conversion system: Haploid strains containing a *sam1-∆Bgl* II*-HOcs* allele at the *SAM1* locus on chromosome XII, a *sam1-∆Sal* I allele at the *HIS3* locus on chromosome XV, and the *sam2::HIS3* allele at the *SAM2* locus on chromosome IV (not pictured) were grown to saturation in YPD supplemented with AdoMet, and plated onto medium lacking AdoMet to select for cells in which a recombination event generates a functional *SAM1* gene and an AdoMet prototrophic cell. The opposite orientations of the *sam1* alleles relative to their centromeres prevents the isolation of single crossovers. Only conversions of the *sam1-∆Bgl* II*-HOcs* allele to wild-type are observed due to the absence of a promoter for the *sam1-∆Sal* I allele. The *sam2::HIS3* allele is missing sufficient information to recombine with *sam1-∆Bgl* II*-HOcs*. Black bars indicate the positions of the mutations. **(B)** Rates of ectopic gene conversion in wild-type and single mutant strains. Rates were determined from a minimum of 10 independent cultures as described in the Methods. Fold decreases (−) and increases (+) from wild-type are indicated in boxes. **(C)** Rates of ectopic gene conversion in *rad27 rad59* double mutant strains. **(D)** Rates of ectopic gene conversion in *rad51::LEU2* and *srs2::TRP1* single mutant, and *rad51::LEU2 rad59-Y92A* and *srs2::TRP1 rad59-Y92A* double mutant strains.

The central strand exchange factor, Rad51 [38], is often required for mechanisms of HR that require *RAD59*, including those involved in spontaneous HR between inverted and unlinked repeat sequences [39,40]. Like *RAD59*, an intact *RAD51* gene is necessary for viability in *rad27::LEU2* mutant cells [18-20], suggesting that *RAD51-*dependent HR plays a critical role in responding to replication lesions. Accordingly, loss of *RAD27* results in increases in HR events that require *RAD51*[18]. We used an assay that measures spontaneous ectopic gene conversion involving unlinked, mutant alleles of the *SAM1* gene [41] to examine effects of the *rad27::LEU2* mutation on HR in haploid strains (Figure 3A). Loss of *RAD27* resulted in a dramatic, 4,700-fold increased rate of ectopic gene conversion (Figure 3B; Additional file 1: Table S2), indicating that accumulation of replication lesions can greatly stimulate HR between unlinked sequences.

The robust stimulatory effect of the loss of the *RAD27* gene on ectopic gene conversion suggested that it could be used for examining the relationship between HR, and growth in the viable *rad27 rad59* double mutants. As observed previously [40], the *rad59::LEU2* mutation conferred a statistically significant 2.7-fold reduction in the rate of ectopic gene conversion (Figure 3B; Additional file 1: Table S2), confirming that *RAD59* plays a role in spontaneous HR between unlinked repeats. While neither the *rad59-K174A* nor *rad59-F180A* mutations had significant effects on their own, they led to significant, 3.1- and 9.3-fold reductions in the stimulatory effect of the *rad27::LEU2* allele in the *rad27::LEU2 rad59-K174A* and *rad27::LEU2 rad59-F180A* double mutants (Figure 3C; Additional file 1: Table S2), suggesting that they confer defects in the utilization of replication lesions by HR.

In contrast to the *rad59-K174A* and *rad59-F180A* mutations, the *rad59-Y92A* mutation caused an 86-fold increased rate of spontaneous ectopic gene conversion (Figure 3B; Additional file 1: Table S2), and, when combined with the *rad27::LEU2* mutation, stimulated the rate of ectopic gene conversion by a statistically significant 7.7-fold over that observed in the *rad27::LEU2* single mutant (Figure 3B and C; Additional file 1: Table S2). The synergistically increased rate of ectopic gene conversion in the *rad27::LEU2 rad59-Y92A* double mutant is consistent with *rad59-Y92A* stimulating HR by a mechanism distinct from the accumulation of replication lesions that results from loss of *RAD27*.

### The hyper-rec effects of the *rad59-Y92A* and *srs2::TRP1* alleles are genetically equivalent

Previous work indicating that *rad59-Y92A* decreases spontaneous *RAD51-*independent HR between directly repeated sequences [27] suggests that the stimulation of ectopic gene conversion is not due to accumulation of recombinogenic lesions. Ectopic gene conversion requires Rad51 to work after lesion formation to catalyze the strand invasion that begins the interaction between unlinked sequences that will repair the lesion [40,42]. If stimulation of HR by *rad59-Y92A* is the result of changes subsequent to Rad51-DNA filament formation, loss of *RAD51* should abolish the stimulatory effect. The rate of ectopic gene conversion in the *rad51::LEU2 rad59-Y92A* double mutant was reduced 50-fold from wild-type, which was nearly identical to the rate in *rad51::LEU2* single mutant cells (Figure 3D; Additional file 1: Table S2). Therefore, stimulation by *rad59-Y92A* requires formation of Rad51-DNA filaments.

Like the *rad59-Y92A* mutation, a null allele of the *SRS2* gene, which encodes a DNA helicase [43] that facilitates the disassembly of Rad51-DNA filaments [36,37], has been shown to stimulate spontaneous gene conversion between non-allelic sequences [44,45]. Consistent with this, we observed a 31-fold increased rate of spontaneous ectopic gene conversion in an *srs2::TRP1* mutant (Figure 3D; Additional file 1: Table S2). As the effects of *srs2::TRP1* and *rad59-Y92A* were similar we examined ectopic gene conversion in the *srs2 rad59-Y92A* double mutant and observed a 38-fold increase over wild-type that was not significantly different from the rates in the *srs2::TRP1* or *rad59-Y92A* single mutants (Figure 3B and 3D; Additional file 1: Table S2). This indicates that *rad59-Y92A* and *srs2::TRP1* are mutually epistatic.

### *rad59-Y92A* is a dominant mutation that stimulates *RAD51*-dependent HR between homologs

**Figure 4:**
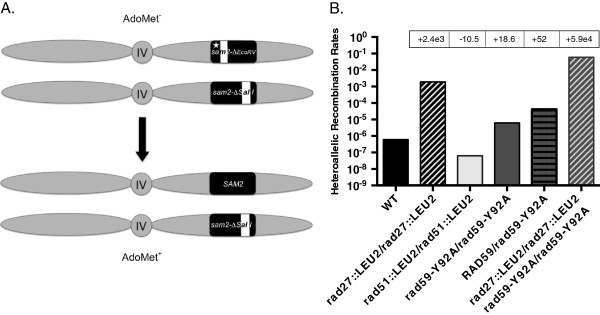
**The *rad59-Y92A *mutation has a dominant, hyper-rec effect on hetero-allelic recombination in diploid strains. (A)** The spontaneous hetero-allelic recombination system: Diploid strains possessing a *sam2-∆EcoR* V*-HOcs* allele at the *SAM2* locus on one copy of chromosome IV, a *sam2-∆SalI* allele on the other copy, and a *sam1::LEU2* allele at the *SAM1* locus on both copies of chromosome XII (not pictured) were grown to saturation in YPD medium supplemented with AdoMet, and plated onto medium lacking AdoMet to select for cells in which a recombination event generates a functional *SAM2* gene and an AdoMet prototrophic cell. Either reciprocal or non-reciprocal recombination events between *sam2-∆EcoR* V*-HOcs* and *sam2-∆Sal* I can generate AdoMet^+^ recombinants. The *sam1::LEU2* allele is missing sufficient information to recombine with the *sam2* alleles. The white bars indicate the positions of the *sam2* mutations. **(B)** Rates of heteroallelic recombination in wild-type, heterozygous and homozygous mutant strains. Rates were determined from a minimum of 10 independent cultures as described in the Methods. Fold decreases (−) and increases (+) from wild-type indicated in boxes*.*

*RAD51-*dependent HR between sister-chromatids and homologous chromosomes are thought to be the primary mechanisms for rescuing lesions, and supporting viability in *rad27* mutant cells [18-20]. One form of inter-homolog HR that requires *RAD51* is recombination between mutant alleles at the same locus, referred to as heteroallelic recombination [46]. Accordingly, the rate of spontaneous recombination between heteroalleles of the *SAM2* gene was reduced 10.5-fold in the *rad51::LEU2/rad51::LEU2* homozygote (Figure 4B; Additional file 1: Table S2). Consistent with its effect on ectopic gene conversion, loss of *RAD27* increased the rate of heteroallelic recombination 2,400-fold, confirming that accumulation of replication lesions robustly stimulates heteroallelic recombination [18].

Similar to its effect on ectopic gene conversion, we observed that *rad59-Y92A* increased the rate of heteroallelic recombination by 19-fold (Figure 4B; Additional file 1: Table S2). Interestingly, the effect of *rad59-Y92A* was dominant with respect to *RAD59*, as the rate in the *RAD59/rad59-Y92A* heterozygote was not significantly different from that in the *rad59-Y92A/rad59-Y92A* homozygote. Like with ectopic gene conversion, combining the *rad27::LEU2* and *rad59-Y92A* alleles in the *rad27/rad27 rad59-Y92A/rad59-Y92A* double homozygote had a synergistic effect on heteroallelic recombination, increasing the rate 25-fold over that observed in the *rad27::LEU2/rad27::LEU2* homozygote. This astonishing, 59,000-fold increased rate of heteroallelic recombination corresponds to a median frequency of recombination where 85% of the surviving colonies are recombinants.

### The *rad59* alleles do not affect a variety of genome destabilizing processes stimulated by the accumulation of replication lesions

Loss of *RAD27* stimulates a variety of mutagenic and clastogenic events [8,16,18,47,48]. The rate of spontaneous mutation at the *CAN1* locus is greatly increased in *rad27::LEU2* mutant cells [8,18,49]. Characterization of these mutations revealed that the majority are short duplications flanked by short, directly repeated sequences that may be created by multiple HR mechanisms [18]. Our data confirm the previous analyses as we observed a 50-fold increased rate of spontaneous mutation at the *CAN1* locus in a *rad27::LEU2* mutant (Table 2; Additional file 1: Table S2). In contrast, the *rad59::LEU2*, *rad59-Y92A, rad59-K174A,* and *rad59-F180A* alleles did not have significant effects on the rate of *CAN1* mutation, nor did the missense alleles have significant effects when combined with the *rad27::LEU2* allele.

**Table 2:** Rates of mutation and unequal sister chromatid recombination in wild-type and mutant haploid strains

**Genotype**	**Mutation rate (10**^**-7**^**)**	**USCR rate (10**^**-6**^**)**
Wild-type	4.0 (3.8, 7.4) [1]	1.0 (0.8, 1.2) [1]
*rad51::LEU2*	n.d.	1.4 (1.0, 1.8) [+1.4]
*rad59::LEU2*	7.5 (6.6, 8.6) [+1.9]	0.82 (0.43, 1.4) [-1.3]
*rad59-Y92A*	4.4 (3.9, 5.3) [+1.1]	1.3 (1.1, 1.8) [+1.3]
*rad59-K174A*	3.2 (1.8, 5.5) [-1.3]	1.1 (0.85, 2.1) [+1.1]
*rad59-F180A*	4.8 (4, 6.9) [+1.2]	0.61 (0.47, 0.95) [-1.6]
*rad27::LEU2*	200 (90, 590) [+50]	47 (39, 100) [+47]
*rad27::LEU2 rad59-Y92A*	220 (60, 510) [+55]	39 (25, 99) [+39]
*rad27::LEU2 rad59-K174A*	130 (110, 190) [+32.5]	38 (33, 53) [+38]
*rad27::LEU2 rad59-F180A*	190 (110, 500) [+47.5]	60 (49, 120) [+60]

Rates of *CAN1* mutation or USCR were determined from at least 10 independent cultures as described in the Methods. The 95% confidence intervals are in parentheses. Fold decreases (−) and increases (+) from wild-type are in brackets. n.d. – not determined.

Loss of *RAD27* has been previously observed to strongly stimulate unequal sister chromatid recombination (USCR) (Additional file 1: Figure S2) [8,50]. We observed a 47-fold increased rate of USCR in *rad27::LEU2* cells (Table 2; Additional file 1: Table S2), confirming the previous results, while loss of *RAD51* had no significant effect. The *rad59::LEU2*, *rad59-Y92A, rad59-K174A,* and *rad59-F180A* alleles did not have significant effects on the rate of USCR, nor did the missense mutations have effects in combination with *rad27::LEU2*, suggesting that *RAD59* does not influence this mechanism of genome rearrangement.

Disrupting lagging strand synthesis by imposing a defect in the processivity of Pol δ, or loss of *RAD27*, was shown previously to substantially increase rates of loss of heterozygosity (LOH) by chromosome loss, and HR between homologs [2,8,10,11,18]. In the present analysis, LOH was examined in diploid strains by simultaneously monitoring changes in the genetic state at three loci on chromosome V (*HXT13*, *CAN1* and *HOM3*) in order to separately determine rates of chromosome loss (reduction to hemizygosity at all three loci), terminal LOH (homozygosity at *HXT13* and *CAN1*), and interstitial LOH (homozygosity at *CAN1*) (Additional file 1: Figure S3; Table 3; Additional file 1: Table S2). Rates of all three events increased substantially in *rad27::LEU2/rad27::LEU2* homozygotes; chromosome loss increased 12-fold, terminal LOH increased 37-fold, and interstitial LOH increased 11-fold, strongly suggesting that replication lesions stimulate HR between homologs when they are repaired, and chromosome loss when they are not. Interestingly, we observed an 18-fold increase in the rate of chromosome loss in *rad51::LEU2/rad51::LEU2* homozygotes, consistent with a requirement for *RAD51* in the rescue of broken chromosomes. In contrast, loss of *RAD51* did not have significant effects on interstitial LOH or terminal LOH, indicating that these inter-chromosomal HR events do not require Rad51.

**Table 3:** Rates of loss of heterozygosity in wild-type and mutant diploid strains

**Genotype**	**ILOH rate (10**^**-5**^**)**	**TLOH rate (10**^**-4**^**)**	**CL rate (10**^**-5**^**)**
Wild-type	2.5 (2.1, 3.1) [1]	0.92 (0.62, 1.2) [1]	3.0 (2.5, 3.9) [1]
*rad51::LEU2/rad51::LEU2*	1.2 (0.92, 2.5) [−2]	1.3 (0.38, 2) [+1.4]	54 (19, 64) [+18]
*rad59::LEU2/rad59::LEU2*	1.8 (1.2, 2.9) [−1.4]	1.4 (1.1, 1.9) [+1.5]	6.2 (5.8, 10.2) [+2]
*rad59-Y92A/rad59-Y92A*	3.2 (2.7, 4.8) [+1.3]	0.95 (0.83, 1.5) [1]	2.5 (2.0, 3.6) [−1.2]
*rad59-K174A*/*rad59-K174A*	2.0 (1.3, 3.5) [−1.3]	0.76 (0.40, 1.1) [−1.2]	5.6 (2.9, 8.4) [+1.9]
*rad59-F180A/rad59-F180A*	3.8 (3.1, 5.1) [+1.5]	0.82 (0.63, 1.7) [−1.1]	3.0 (1.5, 7.9) [1]
*rad27::LEU2/rad27::LEU2*	28 (25, 64) [+11]	34 (24, 47) [+37]	38 (29, 54) [+13]
*rad27::LEU2/rad27::LEU2 rad59-Y92A/rad59-Y92A*	28 (13, 56) [+11]	36 (17, 50) [+39]	29 (23, 74) [+9.7]
*rad27::LEU2/rad27::LEU2 rad59-K174A/rad59-K174A*	26 (22, 55) [+10]	33 (24, 39) [+36]	32 (18, 48) [+11]
*rad27::LEU2/rad27::LEU2 rad59-F180A/rad59-F180A*	52 (29, 76) [+21]	35 (22, 57) [+38]	57 (18,124) [+19]

Rates of interstitial LOH (ILOH), terminal LOH (TLOH), and chromosome loss (CL) from a minimum of 12 independent cultures were determined as described in the Methods. The 95% confidence intervals are in parentheses. Fold decreases (−) and increases (+) from wild-type are in brackets.

As observed above for mutation and USCR (Table 2; Additional file 1: Table S2), the *rad59-Y92A, rad59-K174A*, and *rad59-F180A* alleles had no significant effect on the rates of interstitial LOH, terminal LOH, and chromosome loss in the *rad59/rad59* single mutants, or in the double mutant combinations with the *rad27::LEU2* allele (Table 3; Additional file 1: Table S2). Similarly, *rad59::LEU2* had no significant effect on the rates of interstitial LOH and terminal LOH, but conferred a small (two-fold), statistically significant increase in chromosome loss. These data suggest that *RAD59* has little influence on these mechanisms of LOH*.*

## Discussion

We have explored the role of *RAD59* in mediating responses to DNA lesions that accumulate in *rad27::LEU2* mutant cells, and found that it supports multiple, genetically separable functions. Like the *rad59::LEU2* allele, we found that the *rad59-K166A* allele, which alters a lysine in a conserved, putative α-helical domain (Additional file 1: Figure S1B) [27,34,35], results in synthetic lethality when combined with the *rad27::LEU2* allele (Figure 1). In previous experiments, we found that *rad27::LEU2* mutant cells display a profusion of DSBs [8]. As both *rad59::LEU2* and *rad59-K166A* substantially reduce association of Rad52 with DSBs [21], we speculate that a critical reduction in the association of Rad52 with the many DSBs in *rad27::LEU2 rad59::LEU2* and *rad27::LEU2 rad59-K166A* double mutants may inhibit their rescue by HR, and results in a lethal level of chromosome loss. The *rad59-F180A* and *rad59-K174A* alleles, which change conserved residues in the same α-helical domain altered by *rad59-K166A*, may have incrementally less severe effects on association of Rad52 with DSBs. This may result in their serially reduced inhibition of repair of replication-induced DSBs by HR (Figure 3C; Additional file 1: Table S2) and commensurate effects on growth (Table 1; Additional file 1: Table S2) when combined with *rad27*. An accumulation of *rad27::LEU2 rad59-F180A* double mutant cells in the G2 phase of the cell cycle, as compared to *rad27::LEU2* single mutant or *rad27::LEU2 rad59-K174A* double mutant cells is consistent with more deficient repair of replication-induced DSBs by HR (Figure 3). This further supports the notion that *RAD59* promotes the survival of *rad27::LEU2* mutant cells by facilitating the rescue of replication lesions by HR. Recently, *RAD59* has been shown to be required for the viability of DNA ligase I-deficient mutants, verifying the requirement for this factor in accommodating to incomplete DNA replication [51].

In striking contrast to the other *rad59* alleles, *rad59-Y92A* stimulated HR (Figure 3B; Figure 4B). This hyper-recombinogenic effect was distinct from that caused by *rad27* as it was not accompanied by significant effects on doubling time (Table 1), cell cycle profile (Figure 2), mutation (Table 2), unequal sister chromatid exchange, or LOH (Table 3), suggesting that *rad59-Y92A* does not cause an accumulation of replication lesions. The observation that the stimulatory effect of *rad59-Y92A* was completely suppressed by a null allele of *RAD51*, and was mutually epistatic with a null allele of *SRS2* (Figure 3D), suggests that *rad59-Y92A* may increase HR by increasing the stability of Rad51-DNA filaments, perhaps by changing its interaction with Rad51 (24). An increase in DSBs combined with an increase in the stability of Rad51 filaments at the DSBs may underlay the synergistically increased rates of HR observed in *rad27 rad59-Y92A* double mutants (Figures 3C and 4B). However, since Rad59 also interacts with RPA [52] and RSC [53], the increase in HR observed in *rad59-Y92A* mutant cells may also involve changes in additional processes.

**Figure 5:**
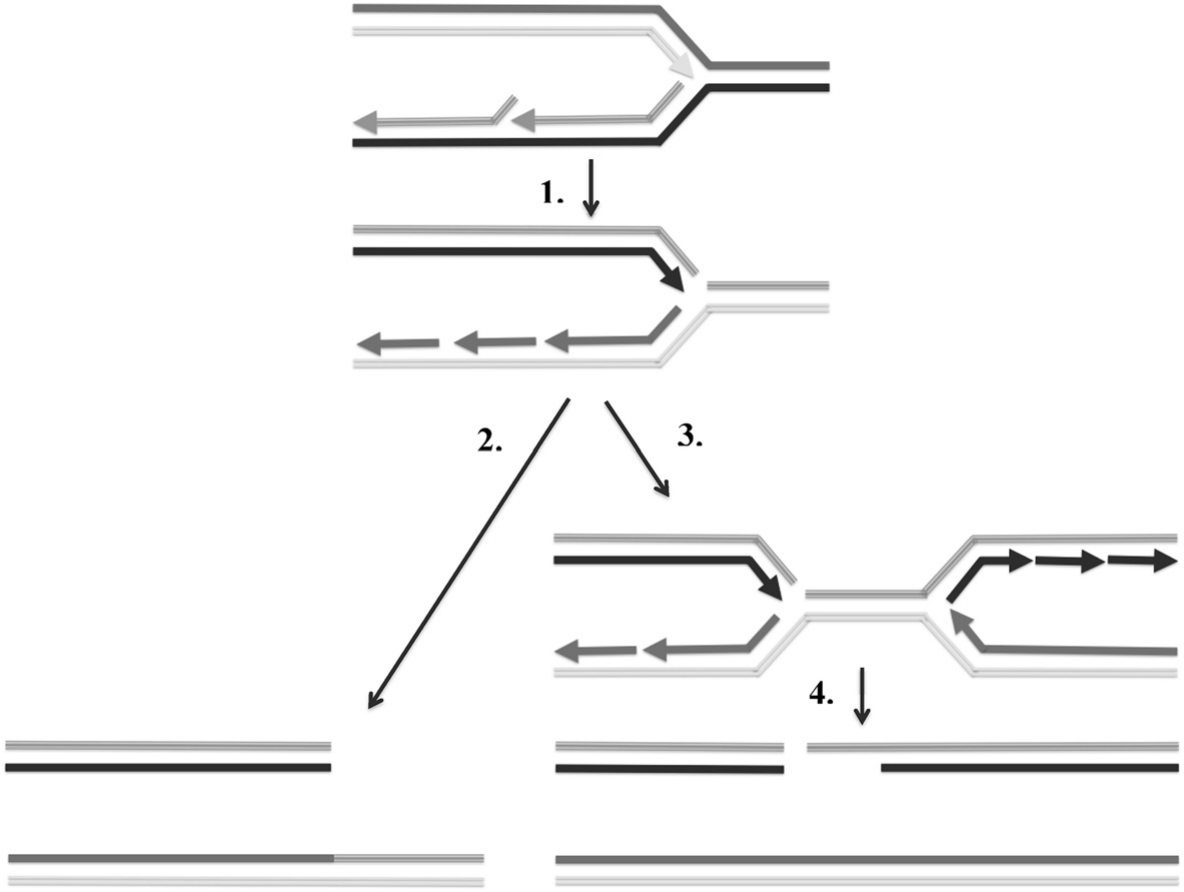
**Models for initiation of *RAD51- *and *RAD59-*dependent and –independent HR by defective lagging strand synthesis.** 1.) Accumulation of daughter strand nicks in the absence of Rad27 nuclease causes replication fork stalling during the next S phase when the lagging strand becomes the template for leading strand synthesis and the replication fork encounters the discontinuity. 2.) The stalled fork is converted into an intact chromatid and a single-ended DSB. The single-ended DSB becomes a substrate for *RAD51-* and *RAD59-*independent HR mechanisms, such as interstitial and terminal LOH (Additional file 1: Figure S3). 3.) The replication fork from an upstream replicon converges with the previously stalled fork. 4.) Converged forks are converted into an intact chromatid and a double-ended DSB. The double-ended DSB becomes a substrate for *RAD51-* and *RAD59-*dependent HR mechanisms, such as ectopic gene conversion and heteroallelic recombination (Figures 3A and 4A).

While our results support a prominent role for *RAD59*-dependent HR in the repair of replication lesions in *rad27::LEU2* mutants, HR mechanisms that do not depend on *RAD59* were also strongly stimulated in *rad27::LEU2* mutants. In particular, inter-chromosomal HR leading to interstitial and terminal LOH events was strongly stimulated by *rad27*, but was unaffected by the *rad59* alleles (Table 3). Inter-chromosomal HR leading to LOH is thought to occur by break-induced replication (BIR) [54]. BIR has been proposed to utilize a single-ended DSB on one homolog to generate a replication fork-like intermediate with the unbroken homolog that may potentially proceed until reaching the end of the donor chromosome (Additional file 1: Figure S4A) [22]. In contrast, *RAD59-*dependent heteroallelic recombination is thought to utilize a double-ended DSB where both ends are rescued, either through concerted interactions with the unbroken homolog, or through the first end interacting with the homolog followed by the second end annealing with the first after gaining sequences copied from the unbroken homolog (Additional; file 1: Figure S4B). The stimulation of both mechanisms of HR between homologs suggests that loss of *RAD27* leads to the accumulation of both single- and double-ended DSBs. DSBs may arise when the failure to remove flaps on the 5′ ends of Okazaki fragments leads to accumulation of nicks on newly replicated lagging strands (Figure 5). Persistence of these nicks into the subsequent cell cycle will leave discontinuities on the template for leading strand synthesis that will stall replication and form single-ended DSBs. If a second replication fork from an adjacent replicon collides with the first stalled fork, a double-ended DSB can arise. A genome-wide increase in replication-induced DSB formation, like that induced by many chemotherapeutic agents, would therefore require a robust response by the HR apparatus to prevent chromosome loss, potentially explaining the critical role of HR in determining sensitivity to these drugs in humans [55,56].

## Conclusions

*RAD59* encodes one of several homologous recombination (HR) factors required for viability of budding yeast cells lacking the DNA replication factor, Rad27. This demonstrates that the HR apparatus is required to prevent the lethal effects of dysfunctional replication, but no link between a specific HR mechanism and survival has been previously established. In this analysis, we show that two *rad59* alleles that diminish association of Rad52 with double-strand breaks are synthetically lethal with *rad27*, while two others coordinately reduce *RAD51-*dependent HR and growth, thus linking *RAD51-*dependent repair with survival. Another allele stimulates HR by stabilizing Rad51-DNA filaments. Therefore, Rad59 influences the repair of replication lesions by HR through its interactions with multiple HR factors. We speculate that the massive increase in replication failure genome-wide that results from loss of Rad27 may be similar to that caused by chemotherapeutic agents in human cells, potentially explaining why the HR apparatus is critical in determining sensitivity to these drugs.

## Methods

### Strains

All strains used in this study were isogenic and are listed in Additional file 1: Table S1. Standard techniques for yeast strain construction and growth were used [57]. Construction of the *rad27::LEU2, rad51::LEU2, rad59::LEU2, rad59-Y92A, rad59-K166A, rad59-K174A, rad59-F180A* and *srs2::TRP1* alleles have been described previously [27,58-60]. The *rad27::LEU2* allele can be followed in crosses by PCR, using the forward primer 5′-GCG TTG ACA GCA TAC ATT-3′, and reverse primer 5′-CGT ACA AAC CAA ATG CGG-3′. The *rad59::LEU2* allele is followed by PCR using the forward primer 5′-GCC ACA GTT TGG CAA GGG-3′, and the reverse primer 5′-GGG TTT GTT GCC ATC TGC G-3′. The *rad59* missense alleles were followed in crosses by allele-specific PCR [27]. Unique forward primers were used to detect *rad59-Y92A* (5′-GCT AAT GAA ACA TTC GGG GC-3′), *rad59-K166A* (5′-AAT GTT ATA ACA GGT CGA AAG C-3′), *rad59-K174A* (5′-AAG GGT TAC GTA GAG GAG AAG-3′), and *rad59-F180A* (5′-AAG AAG GCG TTA TTG AGC GC-3′). All allele-specific PCRs use the same reverse primer (5′-TAT ATA AGT ACG TGA GAT CTA TTT G-3′). Presence of the *rad59*-*K174A* allele is scored by digesting the PCR product with *MseI* restriction endonuclease. DNA was purified for PCR analysis using a standard method [61].

### Synthetic lethality

Diploid yeast strains heterozygous for each of the *rad59* alleles (*rad59/RAD59*) and the *rad27::LEU2* allele (*rad27::LEU2/RAD27*) were sporulated and dissected. After 72 h, five representative tetrads from each diploid were selected. The presence of *rad27* and *rad59* mutant alleles in each of the colonies that arose from the spores was scored using PCR as described above.

### Doubling time

At least 10, five-milliliter YPD (1% yeast extract, 2% peptone, 2% dextrose) cultures were inoculated with colonies arising from the spores of freshly dissected tetrads and grown overnight at 30°. These were sub-cultured into Klett tubes containing five milliliters of YPD medium that were incubated at 30° while shaking. Cell density was measured by monitoring culture turbidity with a Klett-Summerson colorimeter each hour over a 10 h period. Doubling times were calculated using a standard algorithm [62]. The 95% confidence intervals and Mann–Whitney values were determined using the Prism statistics package (GraphPad, La Jolla, CA).

### Flow cytometry

At least five, five-milliliter YPD cultures were inoculated with colonies arising from freshly dissected tetrads and grown overnight at 30°. Overnight cultures were sub-cultured into five milliliters of YPD medium and grown to mid-log phase at 30° defined by growth curve using a Klett-Summerson colorimeter. Cells were processed for flow cytometry using the following adaptation of a published method [63]. The cell density was determined by hemacytometer count and aliquots containing 10^7^ cells were pelleted, resuspended in 70% ice-cold ethanol, and fixed while rotating at 4° overnight. Fixed cells were pelleted, resuspended in 1 ml of citrate buffer (50 mM Na citrate, pH 7.2), and sonicated (Misonix 3000, Farmingdale, NY). Sonicated cells were pelleted, resuspended in citrate buffer and treated with 25 μl of 10 mg/ml RNase A, at 50° for one h, followed by treatment with 50 μl of 20 mg/ml Proteinase K and incubation at 50° for one h. Cells were pelleted and resuspended in 1 ml of citrate buffer, and either rotated overnight at 4°, or stained immediately by adding 16 μl of 1 mg/ml propidium iodide and rotating for 45 min at room temperature in the dark before processing by flow cytometry (Beckman Coulter CyAn ADP 9color, Miami FL). Fractions of cells in the G1, S and G2/M phases of the cell cycle were determined using FlowJo v.7.6.5 image processing software (Tree Star, Ashland, OR). The ratio of cells in G1 vs. S + G2/M were calculated for each trial and the median value for each strain used for comparing cell cycle distributions in different strains. The Mann–Whitney test was used to assess the statistical significance of differences between strains.

### Spontaneous ectopic gene conversion

Spontaneous ectopic gene conversion in haploid strains was assayed as described previously [64], but using substrates described in a separate analysis [41]. All strains contained the *sam1-ΔBgl* II*-HOcs* allele at the *SAM1* locus on chromosome XII, the *sam1-ΔSal* I allele adjacent to the *HIS3* locus on chromosome XV, and a *HIS3* gene replacing the *SAM2* coding sequence at the *SAM2* locus (*sam2::HIS3*) on chromosome IV. The *sam1-∆Bgl* II-HOcs allele has a 117 bp fragment of the *MAT* locus disrupting the *Bgl* II site in the *SAM1* coding sequence, while the *sam1-ΔSal* I allele has a 4 bp insertion at the *Sal* I site [41]. The *sam1-ΔSal* I allele lacks a promoter, preventing conversion events at this locus from generating AdoMet^+^ recombinants. The *sam1-∆Bgl* II-HOcs and *sam1-ΔSal* I alleles are also in opposite orientations relative to their centromeres, preventing the isolation of single crossover recombinants.

At least ten freshly dissected haploid segregants of each strain were inoculated into five-milliliter YPD cultures supplemented with 100 μg/ml of *S*-adenosylmethionine (AdoMet) and grown to saturation at 30°. Appropriate dilutions of each culture were plated onto YPD + AdoMet plates to determine the number of viable cells, and onto YPD plates lacking AdoMet to determine the number of AdoMet prototrophic recombinants.

All rates were determined by the method of the median [65]. Rates and 95% confidence intervals were calculated as described previously [66].

### Spontaneous hetero-allelic recombination

Rates of spontaneous hetero-allelic recombination were determined as for ectopic gene conversion except that different substrates were used in diploid cells. All strains contained the *sam2-ΔEcoR* V*-HOcs* allele at the *SAM2* locus on one copy of chromosome IV, the *sam2-ΔSal* I allele on the other, and a *LEU2* marker replacing the *SAM1* coding sequence at the *SAM1* locus on both copies of chromosome XII. The *sam2-ΔEcoR* V*-HOcs* allele has a 117 bp fragment of the *MAT* locus disrupting the *EcoR* V site, while the *sam2-ΔSal* I allele has a 4 bp insertion disrupting the *Sal* I site [41].

### Mutation rate

Rates of mutation at the *CAN1* locus were examined using a previously published assay [8,10,18]. At least ten freshly dissected segregants were used to inoculate one-milliliter YPD cultures that were grown to saturation at 30°. Appropriate dilutions were plated onto YPD to determine viability and synthetic medium lacking arginine but containing 60 μg/ml of canavanine to select for mutants.

### Unequal sister chromatid recombination (USCR)

Rates of USCR were determined using a previously published assay [8,10,67]. At least ten freshly dissected segregants containing the USCE construct at the *TRP1* locus on chromosome IV and the *his3*∆*200* allele at the *HIS3* locus on chromosome XV, were struck out to single colonies on YPD. After three days of growth at 30°, single colonies were used to inoculate one-milliliter YPD cultures, and grown to saturation at 30°. Appropriate dilutions were plated onto YPD to assess viability and onto medium lacking histidine to determine the number of histidine prototrophic recombinants.

### Loss of heterozygosity (LOH)

Rates of spontaneous LOH by three different mechanisms were assessed using a previously published assay [8]. Freshly dissected haploid segregants containing either the *hxt13::URA3, CAN1,* and *HOM3* alleles, or the *HXT13, can1-100,* and *hom3-10* alleles on chromosome V were crossed and the resulting diploids struck out to single colonies on YPD. At least 12 independent colonies were inoculated into one-milliliter YPD liquid cultures and grown to saturation at 30°. Appropriate dilutions were plated onto YPD for viability and synthetic medium lacking arginine, but containing 60 μg/ml of canavanine to select for clones resistant to canavanine. After three days of growth at 30° canavanine-resistant (Can^R^) colonies were replica plated onto synthetic medium lacking either uracil or threonine to assay for the presence of the *hxt13::URA3* (Ura^+^) and *HOM3* (Thr^+^) alleles, respectively. Rates of interstitial LOH, terminal LOH, and CL were determined from the number of Ura^+^Can^R^Thr^+^, Ura^-^Can^R^Thr^+^, and Ura^-^Can^R^Thr^-^ recombinant colonies, respectively.

### Modeling the Rad59 protein

The crystal structure of the N-terminus of human Rad52 [34] was obtained from the RSCB Protein Data Bank (http://www.rcsb.org/pdb/). This structure was imaged using the molecular modeling program, SYBYL, and the amino acids corresponding to those mutated in the *rad59* missense alleles were identified, and highlighted.

### Availability of supporting data

The data sets supporting the results of this article are included within the article and in Additional file 1.

## Competing interests

The authors declare that they have no competing interests.

## Authors’ contributions

LL carried out the synthetic lethality experiments, LOH genetic studies, flow cytometric analysis, sequence alignment, designed the figures and tables, and drafted the manuscript. GM performed the growth, mutation and USCR rate studies. SO assisted with the synthetic lethality and LOH experiments. BF contributed to the LOH experiments. AB conceived of the study, designed and carried out the ectopic gene conversion and hetero-allelic recombination analyses, and helped draft the manuscript. All authors read and approved the final manuscript.

## Supplementary Material

Additional file 1: Table S1 *Saccharomyces cerevisiae* strains used in this study. **Table S2.** Summary of quantitative data. **Figure S1. A.** Multiple amino acid sequence alignment of ScRad59 with ScRad52 and HsRad52. **B.** Molecular modeling of the proteins encoded by the *rad59* missense alleles demonstrates that Rad59-Y92A is in a different structural motif. **Figure S2.** The unequal sister chromatid recombination (USCR) assay for measuring spontaneous homologous recombination between sister chromatids in haploid yeast. **Figure S3.** The loss of heterozygosity assay for measuring spontaneous Rad51-independent homologous recombination. **Figure S4.** LOH is the recombination product of a single-ended DSB, whereas HAR results from repair of a double-ended DSB. **A)** LOH results from the repair of a single-ended DSB by HR. **B)** HAR results from the repair of a double-ended DSB by HR. Click here for file

## References

[B1] Gordenin DA Malkova AL Peterzen A Kulikov VN Pavlov YI Perkins E Resnick MA Transposon Tn5 excision in yeast: Influence of DNA polymerases α, δ, and ϵ and repair genes Proc Natl Acad Sci U S A 1992 89 3785 3789 10.1073/pnas.89.9.3785 1315039 PMC525575

[B2] Vallen EA Cross FR Mutations in *RAD27* define a potential link between G1 cyclins and DNA replication Mol Cell Biol 1995 15 8 4291 4302 7623823 10.1128/mcb.15.8.4291 PMC230668

[B3] Ruskin B Fink G Mutations in ***POL1*** increase the mitotic instability of tandem inverted repeats in *Saccharomyces cerevisiae* Genetics 1993 133 43 56 8514147 10.1093/genetics/134.1.43 PMC1205442

[B4] Tishkoff DX Boerger AL Bertrand P Filosi N Gaida GM Kane MF Kolodner RD Identification and characterization of *Saccharomyces cerevisiae EXO1*, a gene encoding an exonuclease that interacts with *MSH2* Proc Natl Acad Sci U S A 1997 94 7487 7492 10.1073/pnas.94.14.7487 9207118 PMC23848

[B5] Zou H Rothstein R Holliday junctions accumulate in replication mutants via a RecA homolog-independent mechanism Cell 1997 90 87 96 10.1016/S0092-8674(00)80316-5 9230305

[B6] Chen C Kolodner R Gross chromosomal rearrangements in *Saccharomyces cerevisiae* replication and recombination defective mutants Nat Genet 1999 23 81 85 10471504 10.1038/12687

[B7] Pavlov YI Shcherbakova PV Kunkel TA *In vivo* consequences of putative active site mutations in yeast DNA Polymerases a, ϵ, δ, and ζ Genetics 2001 159 47 64 11560886 10.1093/genetics/159.1.47 PMC1461793

[B8] Navarro MS Bi L Bailis AM A mutant allele of the transcription factor IIH helicase gene, *RAD3*, promotes loss of heterozygosity in response to a DNA replication defect in *Saccharomyces cerevisiae* Genetics 2007 176 1391 1402 10.1534/genetics.107.073056 17483411 PMC1931537

[B9] Venkatesan RN Treuting PM Fuller ED Goldsby RE Norwood TH Gooley TA Ladiges WC Preston BD Loeb LA Mutation at the polymerase active site of mouse DNA polymerase δ increases genomic instability and accerlerates tumorigenesis Mol Cell Biol 2007 27 21 7669 7682 10.1128/MCB.00002-07 17785453 PMC2169052

[B10] Mito E Mokhnatkin JV Steele MC Buettner VL Sommer SS Manthey GM Bailis AM Mutagenic and recombinagenic responses to defective DNA polymerase δ are facilitated by the Rev1 protein in ***pol3-t*** mutants of *Saccharomyces cerevisiae* Genetics 2008 179 1795 1806 10.1534/genetics.108.089821 18711219 PMC2516059

[B11] Galli A Cervelli T Schiestl RH Characterization of the hyperrecombination phenotype of the *pol3-t* mutation of *Saccharomyces cerevisiae* Genetics 2003 164 65 79 12750321 10.1093/genetics/164.1.65 PMC1462548

[B12] Harrington JJ Lieber MR The characterization of a mammalian DNA structure-specific endonuclease EMBO J 1994 13 5 1235 1246 8131753 10.1002/j.1460-2075.1994.tb06373.x PMC394933

[B13] Liu Y Kao HI Bambara RA Flap endonuclease 1: A central component of DNA metabolism Annu Rev Biochem 2004 73 589 615 10.1146/annurev.biochem.73.012803.092453 15189154

[B14] Wu X Wang Z Relationships between yeast Rad27 and Apn1 in response to apurinic/apyrimidinic (AP) sites in DNA Nucleic Acids Res 1999 27 4 956 962 10.1093/nar/27.4.956 9927726 PMC148273

[B15] Tseng HM Tomkinson AE Processing and joining of DNA ends coordinated by interactions among Dnl4/Lif1, Pol4, and FEN-1 J Biol Chem 2004 279 46 47580 47588 10.1074/jbc.M404492200 15342630

[B16] Parenteau J Wellinger RJ Accumulation of single-stranded DNA and destabilization of telomeric repeats in yeast mutant strains carrying a deletion of *RAD27* Mol Cell Biol 1999 19 6 4143 4152 10330154 10.1128/mcb.19.6.4143 PMC104373

[B17] Nowosielska A Bacterial DNA repair genes and their eukaryotic homologues: 5. The role of recombination in DNA repair and genome stability Acta Biochimica Polonica 2007 54 3 483 494 17893749

[B18] Tishkoff DX Filosi N Gaida GM Kolodner RD A novel mutation avoidance mechanism dependent on *S. cerevisiae RAD27* is distinct from DNA mismatch repair Cell 1997 88 253 263 10.1016/S0092-8674(00)81846-2 9008166

[B19] Symington LS Homologous recombination is required for the viability of *rad27* mutants Nucleic Acids Res 1998 26 24 5589 5595 10.1093/nar/26.24.5589 9837987 PMC148039

[B20] Debrauwere H Loeillet S Lin W Lopes J Nicolas A Links between replication and recombination in *Saccharomyces cerevisiae:* a hypersensitive requirement for homologous recombination in the absence of Rad27 activity Proc Natl Acad Sci U S A 2001 98 15 8263 8269 10.1073/pnas.121075598 11459962 PMC37430

[B21] Pannunzio NR Manthey GM Liddell LC Fu BX Roberts CM Bailis AM Rad59 regulates association of Rad52 with DNA double-strand breaks Microbiology Open 2012 1 3 285 297 10.1002/mbo3.31 23170228 PMC3496973

[B22] Paques F Haber JE Multiple pathwyas of recombination induced by double-strand breaks in *Saccharomyces cerevisiae* Micro Mol Biol Rev 1999 63 2 349 404 10.1128/mmbr.63.2.349-404.1999 PMC98970 10357855

[B23] Krogh BO Symington LS Recombination proteins in yeast Annu Rev Genet 2004 38 233 271 10.1146/annurev.genet.38.072902.091500 15568977

[B24] Wu Y Kantake N Sugiyama T Kowalczykowski SC Rad51 protein controls Rad52-mediated DNA annealing J Biol Chem 2008 283 21 14883 14892 10.1074/jbc.M801097200 18337252 PMC2386943

[B25] Davis AP Symington LS The yeast recombinational repair protein Rad59 interacts with Rad52 and stimulates single-strand annealing Genetics 2001 159 515 525 11606529 10.1093/genetics/159.2.515 PMC1461847

[B26] Pannunzio NR Manthey GM Bailis AM *RAD59* is required for efficient repair of simultaneous double-strand breaks resulting in translocations in *Saccharomyces cerevisiae* DNA Repair (Amst) 2008 7 5 788 800 10.1016/j.dnarep.2008.02.003 18373960 PMC2422859

[B27] Pannunzio NR Manthey GM Bailis AM Rad59 and Rad1 cooperate in translocation formation by single-strand annealing in *Saccharomyces cerevisiae* Curr Genet 2010 56 1 87 100 10.1007/s00294-009-0282-6 20012294 PMC2808509

[B28] Sugawara N Ira G Haber JE DNA length dependence of the single-strand annealing pathway and the role of *Saccharomyces cerevisiae RAD59* in double-strand break repair Mol Cell Biol 2000 20 14 5300 5309 10.1128/MCB.20.14.5300-5309.2000 10866686 PMC85979

[B29] Bai Y Symington LS A Rad52 homolog is required for *RAD51*-independent mitotic recombination in *Saccharomyces cerevisiae* Genes Dev 1996 10 16 2025 2037 10.1101/gad.10.16.2025 8769646

[B30] Cortes-Ledesma F Tous C Aguilera A Different genetic requirements for repair of replication-born double-strand breaks by sister-chromatid recombination and break-induced replication Nucleic Acids Res 2007 35 19 6560 6570 10.1093/nar/gkm488 17905819 PMC2095809

[B31] Mott C Symington LS *RAD51-*independent inverted-repeat recombination by a strand-annealing mechanism DNA Repair (Amst) 2011 10 4 408 415 10.1016/j.dnarep.2011.01.007 21317047 PMC3062727

[B32] Cortes-Ledesma F Malagon F Aguilera A A novel yeast mutation, ***rad52-L89F***, causes a specific defect in Rad51-independent recombination that correlates with a reduced ability of ***Rad52-L89F*** to interact with Rad59 Genetics 2004 168 553 557 10.1534/genetics.104.030551 15454565 PMC1448092

[B33] Feng Q During L de Mayolo AA Lettier G Lisby M Erdeniz N Mortensen UH Rothstein R Rad52 and Rad59 exhibit both overlapping and distinct functions DNA Repair (Amst) 2007 6 1 27 37 10.1016/j.dnarep.2006.08.007 16987715

[B34] Kagawa W Kurumizaka H Ishitani R Fukai S Nureki O Shibata T Yokoyama S Crystal structure of the homologous-pairing domain from the human Rad52 recombinase in the undecameric form Mol Cell 2002 10 359 371 10.1016/S1097-2765(02)00587-7 12191481

[B35] Lloyd JA McGrew DA Knight KL Identification of residues important for DNA binding in the full-length human Rad52 protein J Mol Biol 2005 345 2 239 249 10.1016/j.jmb.2004.10.065 15571718

[B36] Veaute X Jeusset J Soustelle C Kowalczykowski SC Fabre F The Srs2 helicase prevents recombination by disrupting Rad51 nucleoprotein filaments Nature 2003 423 309 312 10.1038/nature01585 12748645

[B37] Antony E Tomko EJ Xiao Q Krejci L Lohman TM Ellenberger T Srs2 disassembles Rad51 filaments by a protein-protein interaction triggering ATP turnover and dissociation of Rad51 from DNA Mol Cell 2009 35 1 105 115 10.1016/j.molcel.2009.05.026 19595720 PMC2711036

[B38] Sung P Catalysis of ATP-dependent homologous DNA pairing and strand exchange by yeast RAD51 protein Science 1994 265 5176 1241 1243 10.1126/science.8066464 8066464

[B39] Bai Y Davis AP Symington LS A novel allele of ***RAD52*** that causes severe DNA repair and recombination deficiencies only in the absence of ***RAD51*** or ***RAD59*** Genetics 1999 153 1117 1130 10545446 10.1093/genetics/153.3.1117 PMC1460819

[B40] Jablonovich Z Liefshitz B Steinlauf R Kupiec M Characterization of the role played by the *RAD59* gene of *Saccharoymces cerevisiae* in ectopic recombination Curr Genet 1999 36 13 20 10.1007/s002940050467 10447590

[B41] Bailis AM Maines S Negritto MT The essential helicase gene *RAD3* suppresses short-sequence recombination in *Saccharomyces cerevisiae* Mol Cell Biol 1995 15 5 3998 4008 7623796 10.1128/mcb.15.8.3998 PMC230639

[B42] Liefshitz B Parket A Maya R Kupiec M The role of DNA repair genes in recombination between repeated sequences in yeast Genetics 1995 140 1199 1211 7498763 10.1093/genetics/140.4.1199 PMC1206687

[B43] Rong L Klein HL Purification and characterization of the SRS2 DNA helicase of the yeast *Saccharomyces cerevisiae* J Biol Chem 1993 268 2 1252 1259 8419328

[B44] Rong L Palladino F Aguilera A Klein HL The hyper-gene conversion *hpr5-1* mutation of *Saccharomyces cerervisiae* is an allele of the *SRS2/RADH* gene Genetics 1991 127 75 85 1849857 10.1093/genetics/127.1.75 PMC1204314

[B45] Palladino F Klein HL Analysis of mitotic and meiotic defects in *Saccharomyces cerevisiae SRS2 DNA helicase mutants* Genetics 1992 132 1 23 37 1327956 10.1093/genetics/132.1.23 PMC1205121

[B46] Morrison DP Hastings PJ Characterization of the mutator mutation ***mut5-1.*** Mol Gen Genet 1979 175 1 57 65 10.1007/BF00267856 390308

[B47] Lopes J Ribeyre C Nicolas A Complex minisatellite rearrangements generated in the total or partial absence of Rad27/hFEN1 activity occur in a single generation and are Rad51 and Rad52 dependent Mol Cell Biol 2006 26 17 6675 6689 10.1128/MCB.00649-06 16914748 PMC1592832

[B48] Freudenreich CH Kantrow SM Zakian VA Expansion and length-dependent fragility of CTG repeats in yeast Science 1998 279 853 853 856 9452383 10.1126/science.279.5352.853

[B49] Johnson RE Kovvali GK Prakash L Prakash S Role of yeast Rth1 nuclease and its homologs in mutation avoidance, DNA repair, and DNA replication Curr Genet 1998 34 21 29 10.1007/s002940050362 9683672

[B50] Fasullo MT Davis RW Direction of chromosome rearrangements in *Saccaromyces cerevisiae* by use of *his3* recombinational substrates Mol Cell Biol 1988 8 10 4370 4380 3054515 10.1128/mcb.8.10.4370-4380.1988 PMC365510

[B51] Nguyen HD Becker J Thu YM Costanzo M Koch EN Smith S Myers CL Boone C Bielinsky AK Unligated Okazaki fragments induce PCNA ubiquitnation and a requirement for Rad59-dependent replication fork progression PLoS One 2013 8 6 e66379 10.1371/journal.pone.0066379 23824283 PMC3688925

[B52] Davis AP Symington LS The Rad52-Rad59 complex interacts with Rad51 and replication protein A DNA Repair (Amst) 2003 2 1127 1134 10.1016/S1568-7864(03)00121-6 13679150

[B53] Oum J-H Seong C Kwon Y Ji J-H Sid A Ramakrishnan S Ira G Malkova A Sung P Lee SE Shim EY RSC facilitates Rad59-dependent homologous recombination between sister chromatids by promoting cohesin loading at DNA double-strand breaks Mol Cell Biol 2011 31 19 3924 3937 10.1128/MCB.01269-10 21807899 PMC3187356

[B54] Pohl TJ Nickoloff JA Rad51-independent interchromosomal double-strand break repair by gene conversion requires Rad52 but not Rad55, Rad57, or Dmc1 Mol Cell Biol 2008 28 3 897 906 10.1128/MCB.00524-07 18039855 PMC2223384

[B55] Nikolova T Ensminger M Lobrich M Kaina B Homologous recombination protects mammalian cells from replication-associated DNA double-strand breaks arising in response to methyl methanesulfonate DNA Repair (Amst) 2010 9 10 1050 1063 10.1016/j.dnarep.2010.07.005 20708982

[B56] Nikolova T Hennekes F Bhatti A Kaina B Chloroethylnitrosourea-induced cell death and genotoxicity: cell cycle dependence and the role of DNA double-strand breaks HR and NHEJ. Cell Cycle 2012 11 14 2606 2619 10.4161/cc.20862 22751442

[B57] Sherman F Fink F Hicks J Methods in Yeast Genetics 1986 Cold Spring Harbor, NY: Cold Spring Harbor Laboratory Press

[B58] Schild D Konforti B Perez C Gish W Mortimer RK Isolation and characterization of yeast DNA repair genes. I. Cloning of the *RAD52* gene Curr Genet 1983 7 85 92 10.1007/BF00365631 24173148

[B59] Schild D Calderon IL Contopoulo R Mortimer RK Cloning of yeast recombination repair genes and evidence that several are nonessential genes 1983 New York: Alan R. Liss

[B60] Frank G Qiu J Somsouk M Weng Y Somsouk L Nolan JP Shen B Partial functional deficiency of E160D flap endonuclease-1 mutant *in vitro* and *in vivo* is due to defective cleavage of DNA substrates J Biol Chem 1998 273 49 33064 33072 10.1074/jbc.273.49.33064 9830061

[B61] Hoffman CS Winston F A ten-minute DNA preparation from yeast efficiently releases autonomous plasmids for transformation of *Escherichia coli* Gene 1987 57 2–3 267 272 3319781 10.1016/0378-1119(87)90131-4

[B62] Singleton P Bateria in Biology, Biotechnology, and Medicine 1995 New York: John Wiley & Sons

[B63] Nash N Tokiwa G Anand S Erickson K Futcher AB The *WHI1+* gene of *Saccharomyces cerevisiae* tethers cell division to cell size and is a cyclin homolog EMBO J 1988 7 13 4335 4346 2907481 10.1002/j.1460-2075.1988.tb03332.x PMC455150

[B64] Bailis AM Rothstein R A defect in mismatch repair in *Saccharomyces cerevisiae* stimulates ectopic recombination between homeologous genes by an excision repair dependent process Genetics 1990 126 535 547 2249754 10.1093/genetics/126.3.535 PMC1204210

[B65] Lea DE Coulson CA The distribution of the numbers of mutants in bacterial populations J Genet 1949 49 264 285 10.1007/BF02986080 24536673

[B66] Spell RM Jinks-Robertson S Determination of mitotic recombination rates by fluctuation analysis in *Saccaromyces cerevisiae* Methods Mol Biol 2004 262 3 12 14769952 10.1385/1-59259-761-0:003

[B67] Fasullo MT Davis RW Recombinational substrates designed to study recombination between unique and repetitive sequence *in vivo* Proc Natl Acad Sci U S A 1987 84 6215 6219 10.1073/pnas.84.17.6215 3306671 PMC299041

