# Supplementary material for: Alleles of the homologous recombination gene, RAD59, identify multiple responses to disrupted DNA replication in Saccharomyces cerevisiae
Source: BMC Microbiol. 2013 Oct 14;13:229. doi: 10.1186/1471-2180-13-229 (PMC3852934; doi:10.1186/1471-2180-13-229)
Supplement: Additional file 1: Table S1 — Saccharomyces cerevisiae strains used in this study. Table S2. Summary of quantitative data. Figure S1. A. Multiple amino acid sequence alignment of ScRad59 with ScRad52 and HsRad52. B. Molecular modeling of the proteins encoded by the rad59 missense alleles demonstrates that Rad59-Y92A is in a different structural motif. Figure S2. The unequal sister chromatid recombination (USCR) assay for measuring spontaneous homologous recombination between sister chromatids in haploid yeast. Figure S3. The loss of heterozygosity assay for measuring spontaneous Rad51-independent homologous recombination. Figure S4. LOH is the recombination product of a single-ended DSB, whereas HAR results from repair of a double-ended DSB. A) LOH results from the repair of a single-ended DSB by HR. B) HAR results from the repair of a double-ended DSB by HR. [file 1471-2180-13-229-S1.docx]

**Table S1**  *Saccharomyces cerevisiae* strains used in this study

| **STRAIN** | **GENOTYPE**  **_______** |
| --- | --- |
| ABX2426 | *MAT****a****/α HIS3/his3-URA3-his3 RAD59/rad59::LEU2* |
| ABX2437 | *MAT****a****/α HIS3/his3-URA3-his3 RAD59/rad59-Y92A* |
| ABX2742 | *MAT****a****/α HIS3/his3-URA3-his3 RAD59/rad59-K166A* |
| ABX2429 | *MAT****a****/α TRP1/trp1-1 RAD59/rad59-K174A* |
| ABX2710 | *MAT****a****/α HIS3/his3-URA3-his3 RAD59/rad59-F180A* |
| ABX3138 | *MAT****a****/α HIS3/his3-11, 15 ura3::TRP1/ura3-1 RAD27/rad27::LEU2 RAD59/rad59::LEU2* |
| ABX3334 | *MAT****a****/α HIS3/his3-URA3-his3 RAD27/rad27::LEU2 RAD59/rad59-Y92A* |
| ABX3333 | *MAT****a****/α HIS3/his3-URA3-his3 RAD27/rad27::LEU2 RAD59/rad59-K166A* |
| ABX3336 | *MAT****a****/α HIS3/his3-11, 15 TRP1/trp1-1 RAD27/rad27::LEU2 RAD59/rad59-K174A* |
| ABX3335 | *MAT****a****/α HIS3/his3-URA3-his3 RAD27/rad27::LEU2 RAD59/rad59-F180A* |
| ABX3223 | *MAT****a****/α HIS3-sam1-∆Sal*I*/his3-URA3-his3 ura3::TRP1/ura3-1 SAM1/sam1-∆Bgl*II*-HOcs* *SAM2/sam2::HIS3 RAD27/rad27::LEU2* |
| ABX3243 | *MAT****a****/α HIS3-sam1-∆Sal*I*/his3-URA3-his3 SAM1/sam1-∆Bgl*II*-HOcs* *SAM2/sam2::HIS3 RAD51/rad51::LEU2* |
| ABX3219 | *MAT****a****/α HIS3-sam1-∆Sal*I*/his3-11, 15 ura3::TRP1/ura3::KAN-MX SAM1/sam1-∆Bgl*II*-HOcs* *SAM2/sam2::HIS3 RAD59/rad59::LEU2* |
| ABX3230 | *MAT****a****/α HIS3-sam1-∆Sal*I*/his3-URA3-his3 ura3::TRP1/ura3-1 SAM1/sam1-∆Bgl*II*-HOcs* *SAM2/sam2::HIS3 RAD27/rad27::LEU2 RAD59/rad59-Y92A* |
| ABX3244 | *MAT****a****/α HIS3-sam1-∆SalI/his3-URA3-his3 ura3::TRP1/ura3-1 SAM1/sam1-∆Bgl*II*-HOcs* *SAM2/sam2::HIS3 RAD27/rad27::LEU2 RAD59/rad59-K174A* |
| ABX3224 | *MAT****a****/α HIS3-sam1-∆SalI/his3-URA3-his3 ura3::TRP1/ura3-1 SAM1/sam1-∆Bgl*II*-HOcs* *SAM2/sam2::HIS3 RAD27/rad27::LEU2 RAD59/rad59-F180A* |
| ABX3311 | *MAT****a****/α HIS3-sam1-∆Sal*I*/his3-11, 15 URA3/ura3-1 SAM1/sam1-∆Bgl*II*-HOcs* *SAM2/sam2::HIS3 SRS2/srs2::TRP1* |
| ABX3332 | *MAT****a****/α HIS3-sam1-∆Sal*I*/his3-URA3-his3 SAM1/sam1-∆Bgl*II*-HOcs* *SAM2/sam2::HIS3 RAD59/rad59-Y92A SRS2/srs2::TRP1* |
| ABX3248  ABX3249 | *MAT****a****/α HIS3/his3-11, 15 SAM1/sam1::LEU2* *SAM2/sam2-∆Sal*I  *MAT****a****/α TRP1/trp1-1 SAM1/sam1::LEU2* *SAM2/sam2-∆EcoRV-HOcs* |
| ABX3258  ABX3259  ABX3270  ABX3274  ABX3295  ABX3296 | *MAT****a****/α HIS3/his3-URA3-his3 SAM1/sam1::LEU2* *SAM2/sam2-∆Sal*I *RAD27/rad27::LEU2*  *MAT****a****/α TRP1/trp1-1 SAM1/sam1::LEU2* *SAM2/sam2-∆EcoRV-HOcs RAD27/rad27::LEU2*  *MAT****a****/α HIS3/his3-URA3-his3 SAM1/sam1::LEU2* *SAM2/sam2-∆Sal*I *RAD51/rad51::LEU2*  *MAT****a****/α TRP1/trp1-1 SAM1/sam1::LEU2* *SAM2/sam2-∆EcoRV-HOcs RAD51/rad51::LEU2*  *MAT****a****/α HIS3/his3-URA3-his3 SAM1/sam1::LEU2* *SAM2/sam2-∆Sal*I *RAD59/rad59-Y92A*  *MAT****a****/α TRP1/trp1-1 SAM1/sam1::LEU2* *SAM2/sam2-∆EcoRV-HOcs RAD59/rad59-Y92A* |
| ABX3294 | *MAT****a****/α HIS3/his3-URA3-his3 SAM1/sam1::LEU2* *SAM2/sam2-∆Sal*I *RAD27/rad27::LEU2 RAD59/rad59-Y92A* |
| ABX3292 | *MAT****a****/α HIS3/his3-URA3-his3 SAM1/sam1::LEU2* *SAM2/sam2-∆EcoRV-HOcs RAD27/rad27::LEU2 RAD59/rad59-Y92A* |
| ABX3138 | *MAT****a****/α CAN1/can1-100 ura3::TRP1/ura3-1 RAD27/rad27::LEU2 RAD59/rad59-Y92A* |
| ABX3217 | *MAT****a****/α CAN1/can1-100 TRP1/trp1-1 RAD27/rad27::LEU2 RAD59/rad59-K174A* |
| ABX3161 | *MAT****a****/α HIS3/his3-∆200 trp1-1-his3∆3’-his3-∆5’-URA3/trp1-1 RAD27/rad27::LEU2 RAD59/rad59-Y92A* |
| ABX3159 | *MAT****a****/α his3-∆200/his3-11, 15 TRP1/trp1-1-his3∆3’-his3-∆5’-URA3 RAD27/rad27::LEU2 RAD59/rad59-K174A* |
| ABX3160 | *MAT****a****/α HIS3/his3∆200 trp1-1-his3∆3’-his3-∆5’-URA3/trp1-1 RAD27/rad27::LEU2 RAD59/rad59-F180A* |
| ABX3271 | *MAT****a****/α CAN1/can1-100 HIS3/his3-∆200 trp1-1-his3∆3’-his3-∆5’-URA3/trp1-1 RAD51/rad51::LEU2* |
| ABX1498 | *MAT****a****/α CAN1/can1-100 HIS3/his3-∆Bgl*II*-HOcs TRP1/TRP1 ura3-KANMX/ura3-KANMX HOM3/hom3-10 HXT13/hxt13::URA3* |
| ABX1335 | *MAT****a****/α CAN1/can1-100 HIS3/his3-∆Bgl*II*-HOcs TRP1/TRP1 ura3-KANMX/ura3-KANMX HOM3/hom3-10 HXT13/hxt13::URA3 RAD27/rad27::LEU2* |
| ABX1158 | *MAT****a****/α CAN1/can1-100 HIS3/his3-∆Bgl*II*-HOcs TRP1/trp1-1 ura3-KANMX/ura3-KANMX HOM3/hom3-10 HXT13/hxt13::URA3 RAD51/rad51::LEU2* |
| ABX3220 | *MAT****a****/α CAN1/can1-100 HIS3/HIS3 TRP1/trp1-1 ura3-KANMX/ura3-1 HOM3/hom3-10 HXT13/hxt13::URA3 RAD27/rad27::LEU2 RAD59/rad59-Y92A* |
| ABX3291 | *MAT****a****/α CAN1/can1-100 HIS3/his3-11, 15 TRP1/trp1-1 ura3-KANMX/ura3-1 HOM3/hom3-10 HXT13/hxt13::URA3 LYS2/lys2-∆Bgl*II *RAD27/rad27::LEU2 RAD59/rad59-K174A* |
| ABX3221 | *MAT****a****/α CAN1/can1-100 HIS3/HIS3 TRP1/trp1-1 ura3-KANMX/ura3-1 HOM3/hom3-10 HXT13/hxt13::URA3 RAD27/rad27::LEU2 RAD59/rad59-F180A* |
|  |  |
|  |  |

**^A^** All strains were isogenic and possessed the following alleles unless otherwise noted: *ade2-1 can1-100 his3-11, 15 leu2-3, 112 trp1-1 ura3-1*.

**Table S2** Summary of quantitative data

| **Genotype** | **Assay** | | | | | | | | |
| --- | --- | --- | --- | --- | --- | --- | --- | --- | --- |
|  | **DT ^a^** | **CCD ^b^** | **MR ^c^** | **USCR ^d^** | **ILOH ^e^** | **TLOH ^f^** | **CL ^g^** | **EGC ^h^** | **HAR ^i^** |
| WT | 110.7  (98.7, 120.0) ^j^  [1] ^k^ | 0.193  (0.132,  0.282)  [1] | 4.0x10^-7^  (3.8,  7.4)  [1] | 1.0x10^-6^  (0.8, 1.2)  [1] | 2.5x10^-5^  (2.1, 3.1)  [1] | 9.2x10^-5^  (6.2,  12.0)  [1] | 3.0x10^-5^  (2.5, 3.9)  [1] | 5.0x10^-9^  (2.0, 16.0)  [1] | 6.0x10^-7^  (1.5, 16.0)  [1] |
| *rad27∆* | 164.3  (136.8, 180.0)  [+1.48] | 0.144  (0.081,  0.202)  [-1.3] | 2.0x10^-5^  (0.9, 5.9)  [+50] | 4.7x10^-5^  (3.9, 10.0)  [+47] | 2.8x10^-4^  (2.7, 4.8)  [+11] | 3.4x10^-3^  (2.4, 4.7)  [+37] | 3.8x10^-4^  (2.9, 5.4)  [+13] | 2.4x10^-5^  (1.3, 5.3)  [+4700] | 1.4x10^-3^  (0.7, 4.0)  [+2400] |
| *rad51∆* | n.d. ^l^ | n.d. | n.d. | 1.4x10^-6^  (1.0, 1.8)  [+1.4] | 1.2x10^-5^  (0.92, 2.5)  [-2] | 1.3x10^-4^  (0.38,  2.0)  [+1.4] | 5.4x10^-4^  (1.9, 6.4)  [+18] | 1.1x10^-10^  (0.9,  3.3)  [-47] | 5.5x10^-8^  (3.6,  17.4)  [-11] |
| *rad59∆* | n.d. | n.d. | 7.5x10^-7^  (6.6, 8.6)  [+1.9] | 8.2x10^-7^  (4.3, 14.0)  [-1.3] | 1.8x10^-5^  (1.1, 2.9)  [-1.4] | 1.4x10^-4^  (1.1, 1.9)  [+1.5] | 6.2x10^-5^  (5.8, 10.2)  [+2.0] | 2.0x10^-9^  (1.0, 4.0)  [-2.5] | n.d. |
| *r59-Y92* | 118.7  (96.7, 123.6)  [+1.07] | 0.198  (0.130,  0.305)  [1] | 4.4x10^-7^  (3.9, 5.3)  [+1.1] | 1.3x10^-6^  (1.1, 1.8)  [+1.3] | 3.2x10^-5^  (2.7, 4.8)  [+1.3] | 9.5x10^-5^  (8.3,  15.0)  [1] | 2.5x10^-5^  (2.0,  3.6)  [-1.2] | 4.3x10^-7^  (1.0, 16.5)  [+86] | 5.4x10^-6^  (1.7,  21.7)  [+19] |
| *RAD59/*  *rad59-Y* | n.d. | n.d. | n.d. | n.d. | n.d. | n.d. | n.d. | n.d. | 3.1x10^-5^  (0.7, 5.8)  [+52] |
| *r59-K174* | 131.1  (110.8, 146.7)  [1.18] | n.d. | 3.2x10^-7^  (1.8, 5.5)  [-1.3] | 1.1x10^-6^  (0.85, 2.1)  [+1.1] | 2.0x10^-5^  (1.3,  3.5)  [-1.3] | 7.6x10^-5^  (4.0, 11.0)  [-1.2] | 5.6x10^-5^  (2.9, 8.4)  [+1.9] | 3.0x10^-9^  (1.1, 15.0)  [-1.6] | n.d. |
| *r59-F180* | 112.3  (98.8, 128.0)  [+1.02] | 0.173  (0.132,  0.209)  [-1.1] | 4.8x10^-7^  (4.0, 6.9)  [+1.2] | 6.1x10^-7^  (4.7, 9.5)  [-1.6] | 3.8x10^-5^  (2.1,  5.1)  [+1.5] | 8.2x10^-5^  (6.3,  17.0)  [-1.1] | 3.0x10^-5^  (1.5, 7.9)  [1] | 1.2x10^-8^  (0.3, 1.5)  [+2.4] | n.d. |
| *srs2∆* | n.d. | n.d. | n.d. | n.d. | n.d. | n.d. | n.d. | 1.6x10^-7^  (0.8,  7.5)  [+31] | n.d. |
| *r27∆*  *r59-Y92* | 176.0  (135.6, 195.2)  [+1.59] | 0.152  (0.079,  0.267)  [-1.3] | 2.2x10^-5^  (0.6, 5.1)  [+55] | 3.9x10^-5^  (2.5, 9.9)  [+39] | 2.8x10^-5^  (1.3,  5.6)  [+11] | 3.6x10^-3^  (1.7, 5.0)  [+39] | 2.9x10^-4^  (2.3, 7.4)  [+10] | 1.8x10^-4^  (0.9, 6.9)  [+36000] | 5.9x10^-2^  (1.4,  6.5)  [+59000] |
| *r27∆*  *r59-K174* | 152.5  (126.3,  177.1)  [+1.38] | n.d. | 1.3x10^-5^  (1.1, 1.9)  [+33] | 3.8x10^-5^  (3.3, 5.3)  [+38] | 2.6x10^-5^  (2.2,  5.5)  [+10] | 3.3x10^-3^  (2.4,  3.9)  [+36] | 3.2x10^-4^  (1.8,  4.8)  [+11] | 7.4x10^-6^  (0.3,  11.2)  [+1488] | n.d. |
| *r27∆*  *r59-F180* | 204.9  (182.9,  230.3)  [+1.85] | 0.056  (0.021,  0.083)  [-3.5] | 1.9x10^-5^  (1.1, 5.0)  [+48] | 6.0x10^-5^  (4.9, 12.0)  [+60] | 5.2x10^-5^  (2.9, 7.6)  [+21] | 3.5x10^-3^  (2.2,  5.7)  [+38] | 5.7x10^-4^  (1.8, 12.4)  [+19] | 2.5x10^-6^  (0.6,  9.1)  [+504] | n.d. |
| *srs2∆*  *r59-Y92* | n.d. | n.d. | n.d. | n.d. | n.d. | n.d. | n.d. | 1.9x10^-7^  (0.8,  4.3)  [+38] | n.d. |
| *rad51∆ r59-Y92* | n.d. | n.d. | n.d. | n.d. | n.d. | n.d. | n.d. | 1.0x10^-10^  (0.8,  3.0)  [-50] | n.d. |

^a^ Doubling time (minutes)

^b^ Cell cycle distribution (ratio of cells in G1 vs. S + G2/M)

^c^ Mutation rate (events/cell/generation)

^d^ Unequal sister chromatid recombination rate (events/cell/generation)

^e^ Interstitial loss of heterozygosity rate (events/cell/generation)

^f^ Terminal loss of heterozygosity rate (events/cell/generation)

^g^ Chromosome loss rate (events/cell/generation)

^h^ Ectopic gene conversion rate (events/cell/generation)

^i^ Heteroallelic recombination rate (events/cell/generation)

^j^ 95% confidence interval in parentheses

^k^ Fold difference from wild-type in brackets

^l^ not determined


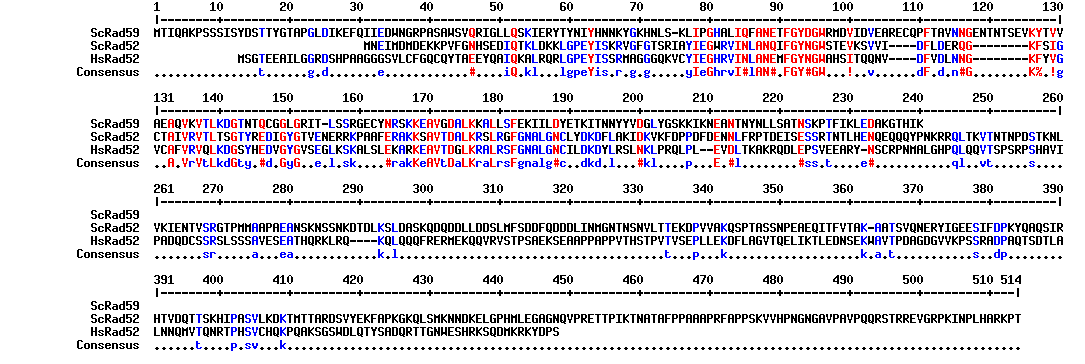


B.

A.


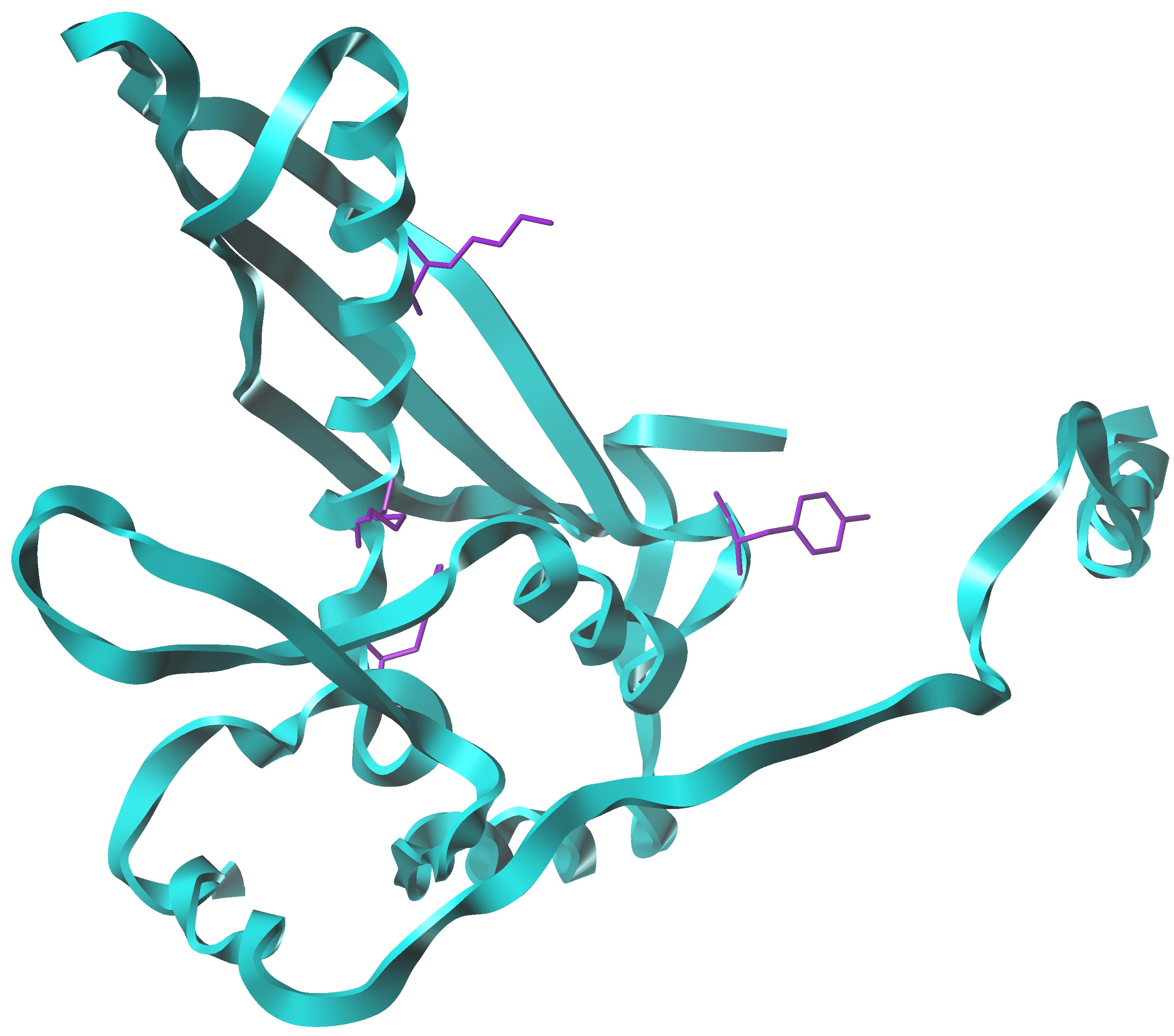


Y81 **[Y92]**

K144 [**K166]**

K152 **[K174]**

F158 **[F180]**

**Figure S1**

**A. Multiple amino acid sequence alignment of ScRad59 with ScRad52 and HsRad52.** The ScRad59, ScRad52, and HsRad52 amino acid sequences were aligned using the MutlAlin multiple sequence alignment interface (<http://multalin.toulouse.inra.fr/multalin/>). Identical and conserved residues are highlighted in grey. Amino acid residues corresponding to the *rad59* missense alleles are boxed (*rad59-Y92A, rad59-K166A, rad59-K174A,* and *rad59-F180A)*. The Rad59-Y92 residue is starred, indicating that it is located in a different structural motif.

**B. Molecular modeling of the proteins encoded by the *rad59* missense alleles demonstrates that Rad59-Y92A is in a different structural motif.** The ScRad59 protein structure was modeled from a monomer of the crystalized ring structure of HsRad52, using the RSCB Protein Data Bank (<http://www.rcsb.org/pdb/>) and molecular modeling program, SYBYL. The HsRad52 amino acid residues analogous to the *rad59* missense alleles (pink rods) are labeled in plain text next to their corresponding ScRad59 residues (bold). The residues equivalent to ScRad59-K166, -K174, and -F180 are located on the same alpha helical motif, while the residue analogous to ScRad59-Y92 is positioned on a separate loop motif (starred).


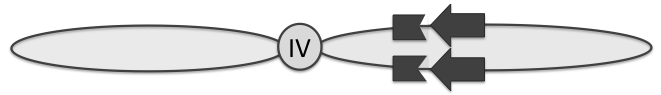

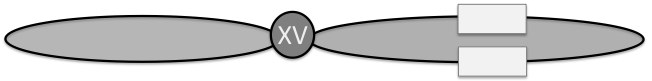

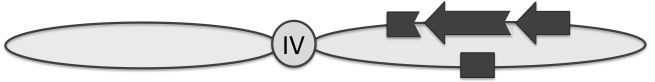

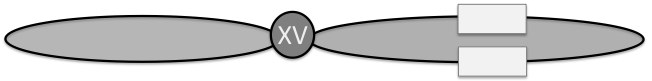


*Δ5’*

*his3Δ3’*

*his3Δ200*

*HIS3*

*Δ5’*

*his3Δ3’*

*his3Δ200*

**Figure S2 The unequal sister chromatid recombination (USCR) assay for measuring spontaneous homologous recombination between sister chromatids in haploid yeast.** The recombination substrates utilized in this assay were previously described (Fasullo and [Davis 1987](#_ENREF_1))*,* and include 5’ and 3’ truncated alleles of the *HIS3* gene, positioned head-to-tail at the *TRP1* locus on chromosome IV. The native *HIS3* sequence on chromosome XV is disrupted (*his3*∆*200)* so it cannot be used as a template for repair. The *his3* alleles share 300 bp of homologous sequence. Recombination between *his3-∆3’* on one chromatid and *his3-∆5’* on the other chromatid generates an intact *HIS3* allele and a histidine prototrophic cell.

**Figure S3. The loss of heterozygosity assay for measuring spontaneous Rad51-independent homologous recombination.** Spontaneous loss of heterozygosity (LOH) by several mechanisms was determined in diploid yeast. One chromosome contains the *hxt13::URA3, CAN1,* and *HOM3* alleles at the *HXT13*, *CAN1*, and *CAN1* loci on chromosome V, while the other carries *HXT13, can1-100,* and *hom3-10.* The primary genetic event examined by this assay is loss of the wild-type allele of *CAN1* by either chromosome loss or recombination with the *can1-100* allele on the homolog resulting in resistance to canavanine. Three distinct events are identified: 1.) Interstitial LOH (ILOH), where the HR machinery generates two copies of *can1-100* but leaves the *HXT13* and *HOM3* loci undisturbed, 2.) Terminal LOH (TLOH), where the HR machinery generates homozygosity for both *can1-100*  and *HXT13*, or 3.) Chromosome Loss (CL), where the copy of chromosome V carrying the *hxt13::URA3*, *CAN1*, and *HOM3* alleles is lost. These events can be distinguished phenotypically by the presence or absence of the selectable markers at the *HXT13, CAN1*, and *HOM3* loci: ILOH (Ura^+^Can^R^Thr^+^), TLOH (Ura^-^Can^R^Thr^+^) or CL (Ura^-^Can^R^Thr^-^).


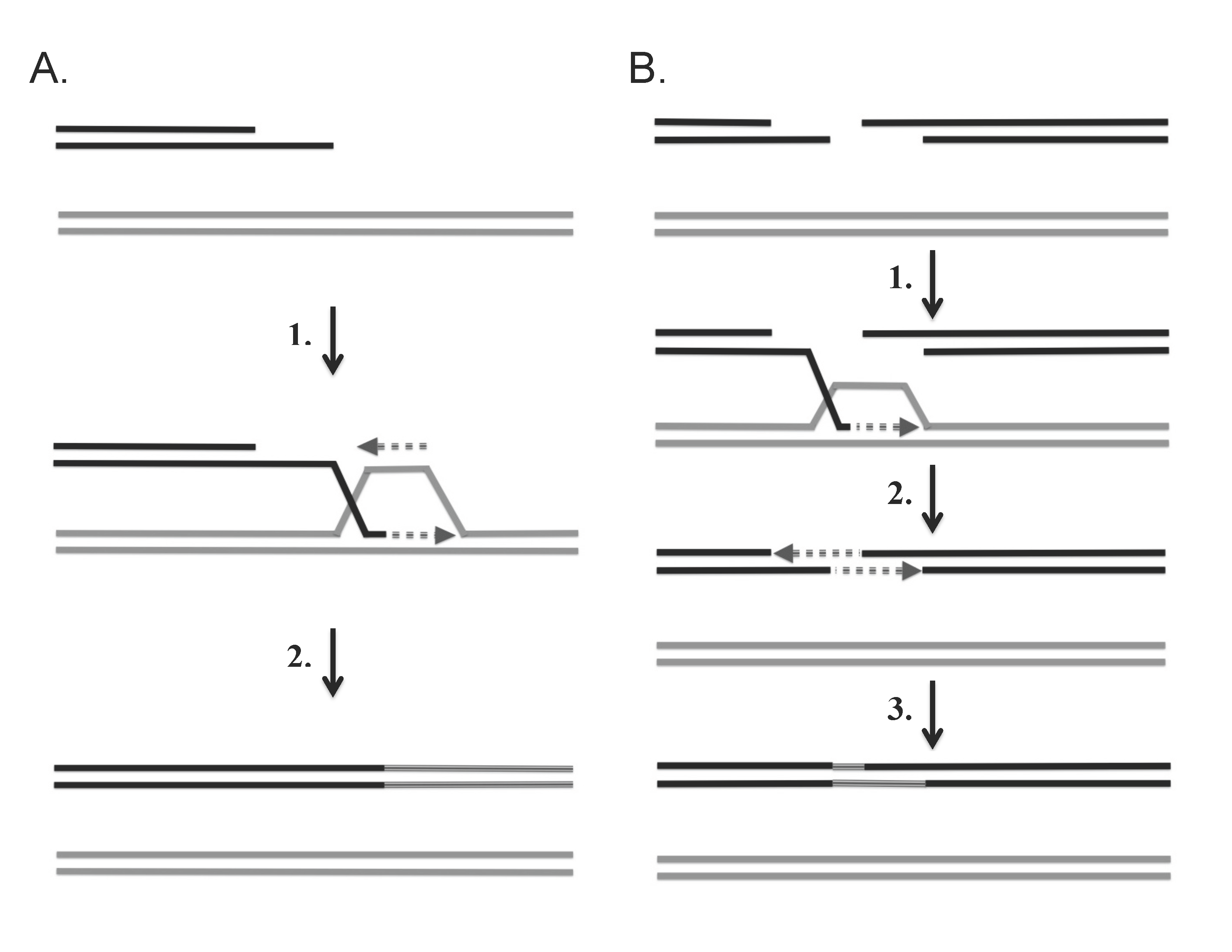
 **Figure S4 LOH is the recombination product of a single-ended DSB, whereas HAR results from repair of a double-ended DSB.**

**A) LOH results from the repair of a single-ended DSB by HR** 1.) One end of a broken chromosome forms a heteroduplex with homologous sequences on the intact homologous chromosome. DNA synthesis initiated from the heteroduplex generates a replication fork-like intermediate, where both strands of the donor chromosome are copied. 2.) DNA synthesis proceeds until the intermediate encounters a converging replication fork, centromere, or the end of the donor chromosome. This generates an intact chromosome that shares complete sequence identity with the homolog within the copied region.

**B) HAR results from the repair of a double-ended DSB by HR** 1.) One end of a broken chromosome forms a heteroduplex with homologous sequences on the intact homologous chromosome. Heteroduplex formation displaces the strand of the unbroken chromosome with the same polarity. Extension of the invading strand by DNA synthesis using the homologous chromosome as the template restores information lost from the broken chromosome. 2.) One potential mechanism for rescuing the second end of the broken chromosome is for the second end to anneal with the first end once DNA synthesis has restored a sufficient length of DNA. DNA synthesis from both ends of the broken chromosome restores the missing information, using the complementary strands as templates. 3.) Ligation of nicked strands produces an intact chromosome once DNA synthesis is complete. The amount of information that may be transferred to the broken homolog is determined by the amount of DNA copied from the unbroken homolog.
